# A Clique-Based Separator for Intersection Graphs of Geodesic Disks in $\mathbb {R}^2$

**DOI:** 10.1007/s00453-025-01337-5

**Published:** 2025-08-29

**Authors:** Boris Aronov, Mark de Berg, Leonidas Theocharous

**Affiliations:** 1 Department of Computer Science and Engineering, Tandon School of Engineering, New York University, Brooklyn, NY 11201 USA; 2 Department of Mathematics and Computer Science, TU Eindhoven, Eindhoven, The Netherlands; 3 School of Electrical Engineering and Computer Science, University of Ottawa, Ottawa, Canada

**Keywords:** Computational geometry, Intersection graphs, Separator theorems

## Abstract

Let *d* be a (well-behaved) shortest-path metric defined on a path-connected subset of $\mathbb {R}^2$ and let $\mathcal {D}=\{D_1,\ldots,D_n\}$ be a set of geodesic disks with respect to the metric *d*. We prove that $\mathcal {G}^{\times }(\mathcal {D})$, the intersection graph of the disks in $\mathcal {D}$, has a clique-based separator consisting of $O(n^{3/4+\varepsilon })$ cliques. This significantly extends the class of objects whose intersection graphs have small clique-based separators. Our clique-based separator yields an algorithm for *q*-Coloring that runs in time $2^{O(n^{3/4+\varepsilon })}$, assuming the boundaries of the disks $D_i$ can be computed in polynomial time. We also use our clique-based separator to obtain a simple, efficient, and almost exact distance oracle for intersection graphs of geodesic disks. Our distance oracle uses $O(n^{7/4+\varepsilon })$ storage and can report the hop distance between any two nodes in $\mathcal {G}^{\times }(\mathcal {D})$ in $O(n^{3/4+\varepsilon })$ time, up to an additive error of one. So far, distance oracles with an additive error of one that use subquadratic storage and sublinear query time were not known for such general graph classes.

## Introduction

**(Clique-based) separators.** The Planar Separator Theorem states that any planar graph with *n* nodes has a *balanced separator* of size $O(\sqrt{n})$. In other words, for any planar graph $\mathcal {G}=(V,E)$ there exists a subset $S\subset V$ of size $O(\sqrt{n})$ with the following propertyautoedited,:^1^$V\setminus S$ can be split into subsets *A* and *B* with $|A|\leqslant 2n/3$ and $|B|\leqslant 2n/3$ such that there are no edges between *A* and *B*. This fundamental result was first proved in 1979 by Lipton and Tarjan [22] and has been simplified and refined in several ways, see, e.g., [13, 14]. The Planar Separator Theorem proved to be extremely useful for obtaining efficient divide-and-conquer algorithms for a large variety of problems on planar graphs.

In this paper we are interested in *geometric intersection graphs* in the plane. These are graphs whose node set corresponds to a set $\mathcal {D}$ of objects in the plane and that have an edge between two nodes iff the corresponding objects intersect. We will denote this intersection graph by $\mathcal {G}^{\times }(\mathcal {D})$. *(Unit) disk graphs* and *string graphs*—where the set $\mathcal {D}$ consists of (unit) disks and curves, respectively—are among the most popular types of intersection graphs. Unit disk graphs in particular have been studied extensively, because they serve as a model for wireless communication networks. It is well known that disk graphs are a generalization of planar graphs, because by the Circle Packing Theorem (also known as the Koebe–Andreev–Thurston Theorem) every planar graph is the intersection graph of a set of disks with disjoint interiors [27]. Because of the widespread use of the Planar Separator Theorem, it is therefore natural to try to extend it to intersection graphs. A direct generalization is clearly impossible, however, since intersection graphs can contain arbitrarily large cliques.

There are several ways to still obtain separator theorems for intersection graphs. One can allow the size of the separator to depend on *m*, the number of edges, instead of on the number of vertices. For example, any string graph admits a separator of size $O(\sqrt{m})$ [20]. Note that in the worst case, when $m=\Theta (n^2)$, this result is not very useful. One may also put restrictions on the set $\mathcal {D}$ to prevent large cliques. For example, if we restrict the *density*^2^ of a set $\mathcal {D}$ of planar objects to some value $\lambda$, then we can obtain a separator^3^ of size $O(\sqrt{\lambda n})$ [17]. This result implies that intersection graphs of disks (or, more generally, of *fat* objects) whose ply is bounded by a constant admit a separator of size $O(\sqrt{n})$. (The *ply* of a set of objects in $\mathbb {R}^2$ is the maximum, over all points $q\in \mathbb {R}^2$, of the number of objects containing *q*.) In the worst case, however, the ply can be *n*, in which case the result is not useful. A novel approach by De Berg et al. [11], which generalizes earlier work of Fu [15], uses what we will call *clique-based separators*. A clique-based separator is a (balanced) separator consisting of cliques, and its size is not measured as the total number of nodes of the cliques, but as the number of cliques. De Berg et al. showed that the intersection graph of a set $\mathcal {D}$ of convex fat object in the plane admits a separator consisting of $O(\sqrt{n})$ cliques. In fact, their result is slightly stronger: it states that there is a separator *S* that can be decomposed into $O(\sqrt{n})$ cliques $C_1,\ldots,C_t$ such that $\sum _{i=1}^t \gamma (|C_i|) = O(\sqrt{n})$, where $\gamma (x)=\log x$ is a cost function^4^ on the cliques. Note that this result holds irrespective of the ply of the objects. Clique-based separators provide a powerful tool, because cliques can be handled efficiently for many problems. Indeed, De Berg et al. used their clique-based separators to develop a unified framework to solve many classic graph problems, including Independent Set, Dominating Set, Feedback Vertex Set, and more. Clique-based separators have been used to solve other problems as well, such approximating the diameter of a unit-disk graph [7] and computing list homomorphisms [18]. They can also be used to design distance oracles, as discussed in more detail later. To expand the applicability of this powerful tool, we would like to find clique-based separators of sublinear size for other classes of objects (besides fat convex objects, as studied in [11]) as well.

Recently De Berg et al. [12] proved clique-based separator theorems for map graphs and intersection graphs of pseudo-disks, which admit clique-based separators consisting of $O(\sqrt{n})$ and $O(n^{2/3})$ cliques, respectively. They also showed that intersection graphs of *geodesic disks inside a simple polygon*—that is, geodesic disks induced by the standard shortest-path metric inside the polygon—admit a clique-based separator consisting of $O(n^{2/3})$ cliques. They left the case of geodesic disks in a polygon with holes as an open problem. We note that string graphs, which subsume the class of intersection graphs of geodesic disks, do *not* admit clique-based separators of sublinear size, since string graphs can contain arbitrarily large induced bipartite cliques.

**Our results.** In this paper we show that intersection graphs of geodesic disks in a polygon with holes admit a clique-based separator consisting of a sublinear number of cliques. Our result is actually much more general, as it shows that *for any well-behaved shortest-path metric d* defined on a path-connected and closed subset $F\subset \mathbb {R}^2$, a set of geodesic disks with respect to that metric admits a separator consisting of $O(n^{3/4+\varepsilon })$ cliques. This includes the shortest-path metric defined by a set of (possibly curved) obstacles in the plane, the shortest-path metric defined on a terrain, and the shortest-path metric among weighted regions in the plane. (See Sect. 2.3 for the formal requirements on a well-behaved shortest-path metric.) Note that we do not require shortest paths to be unique, nor do we put a bound on the number of intersections between the boundaries of two geodesic disks, nor do we require the metric space to have bounded doubling dimension.

The generality of our setting implies that previous approaches to construct clique-based separators will not work. For example, the method of De Berg et al. [11] to construct a clique-based separator for Euclidean disks crucially relies on disks being fat objects. More precisely, it uses the fact that many relatively large fat objects intersecting a given square must form *O*(1) cliques. This property already fails for geodesic disks for the Euclidean shortest-path metric inside a simple polygon. Thus, De Berg et al. [12] use a different approach: they prove that geodesic disks inside a simple polygon are *pseudo-disks* and then show how to obtain a clique-based separator for pseudo-disks. In our more general setting, however, geodesic disks need not be pseudo-disks: the boundaries of two geodesic disks in an arbitrary metric can intersect arbitrarily many times—this is already the case for the Euclidean shortest-path metric in a polygon with holes. Thus we have to proceed differently.

The idea of our new approach is as follows. We first reduce the ply of the set $\mathcal {D}$ by removing all cliques of size $\Omega (n^{1/5})$, thus obtaining a set $\mathcal {D}^*$ of ply $O(n^{1/5})$. (The removed cliques will eventually be added to the separator. A similar preprocessing step to reduce the ply was used in [12] to handle pseudo-disks.) The remaining arrangement can still be arbitrarily complex, however. To overcome this, we ignore the arrangement induced by the disks, and instead focus on the realization of the graph $\mathcal {G}^{\times }(\mathcal {D}^*)$ obtained by drawing a shortest path $\pi _{ij}$ between the centers of any two intersecting disks $D_i,D_j\in \mathcal {D}^*$. We then prove that the number of edges of $\mathcal {G}^{\times }(\mathcal {D}^*)$ must be $O(n^{8/5})$; otherwise there will be an intersection point of two shortest paths $\pi _{ij},\pi _{k\ell }$ that has large ply, which is not possible due to the preprocessing step. Since the number of edges of $\mathcal {G}^{\times }(\mathcal {D}^*)$ is $O(n^{8/5})$ we can use the separator result on string graphs to obtain a separator of size $O(\sqrt{n^{8/5}}) = O(n^{4/5})$. Adding the cliques that were removed in the preprocessing step then yields a separator consisting of $O(n^{4/5})$ cliques and singletons. Finally, we devise a bootstrapping mechanism to further decrease the size of the separator, leading to a separator consisting of $O(n^{3/4+\varepsilon })$ cliques.

Clique-based separators give sub-exponential algorithms for Maximum Independent Set, Feedback Vertex Set, and *q*-Coloring for constant *q* [12]. When using our clique-based separator, the running times for Maximum Independent Set and Feedback Vertex Set are inferior to what is known for string graphs. For *q*-Coloring with $q\geqslant 4$ this is not the case, however, since 4-Coloring does not admit a sub-exponential algorithm, assuming eth [5]. Our clique-based separator, on the other hand, yields an algorithm with running time $2^{O(n^{3/4+\varepsilon })}$, assuming the boundaries of the disks $D_i$ can be computed in polynomial time. Another application of our separator result is to distance oracles, as discussed next.

**Application to distance oracles.** One of the most basic queries one can ask about a (possibly edge-weighted) graph $G=(V,E)$ is a *distance query*: given two nodes $s,t\in V$, what is the distance between them? A data structure answering such queries is called a *distance oracle*. One can simply store all pairwise distances in a distance matrix so that distance queries can be answered in *O*(1) time, but this requires $\Theta (n^2)$ storage, where $n:=|V|$. At the other extreme, one can do no preprocessing and answer a query by running a single-source shortest path algorithm; this uses $O(n+m)$ storage, where $m:=|E|$ and a query costs $\Omega (n)$ time. The main question is if sublinear query time can be achieved with subquadratic storage.

As an application of our clique-based separator we present a simple and almost exact distance oracle for intersection graphs of geodesic disks. While our approach is standard—it was already used in 1996 by Arikati et al. [3]—it does provide an almost exact distance oracle in a more general setting than what was known before, thus showing the power of clique-based separators. Our distance oracle uses $O(n^{7/4+\varepsilon })$ storage and can report the hop distance between any two nodes in $\mathcal {G}^{\times }(\mathcal {D})$ in $O(n^{3/4+\varepsilon })$ time, up to an additive error of one. This is the first distance oracle with only an additive error for a graph class that is more general than planar graphs: even for Euclidean unit-disk graphs the known distance oracle has a multiplicative error [6, 16]. (Admittedly, the storage and query time of this oracle are significantly better than of ours.) We note that clique-based separators were recently also used (in a similar way) to obtain a distance oracle for so-called transmission graphs [10]. (A transmission graph is a directed graph whose nodes correspond to Euclidean disks in the plane, and that have an edge from disk $D_i$ to disk $D_j$ iff the center of $D_j$ is contained in $D_i$.)

**Related work on distance oracles.** There has been a lot of work on distance oracles, both for undirected and for directed graphs; see for example the survey by Sommer [25] for an overview of the work up to 2014. Since our interest is in intersection graphs, which are undirected, hereafter we will restrict our discussion to undirected graphs.

In the edge-weighted setting, one obviously needs to store all edge weights to answer queries exactly. Hence, for exact distance oracles on weighted graphs the focus has been on on sparse graphs and, in particular, on planar graphs. Very recently, Charalampopoulos et al. [8] achieved a major breakthrough by providing an exact distance oracle for planar graphs that uses $O(n^{1+o(1)})$ storage and has $O(\log ^{2+o(1)})$ query time. The solution even works for weighted planar graphs, and it allows other trade-offs as well. For $(1+\varepsilon )$-approximate distance oracles on planar graphs, Le and Wulff-Nilsen [19] showed how to achieve $O(1/\varepsilon ^2)$ query time with $O(n/\varepsilon ^2)$ storage. We refer the reader to the recent paper by Charalampopoulos et al. [8] for a historical overview of the results on planar distance oracles.

For non-planar graphs the results are far less good: the distance oracles are approximate and typically use significantly super-linear storage; see the survey by Sommer [25]. For example, Chechik [9] presented a $(2k-1)$-approximate distance oracle using $O(kn^{1+1/k})$ storage and with *O*(1) query time, for any given integer $k\geqslant 1$. Moreover, for $t<2k-1$, any *t*-approximate distance oracle requires $\Omega (kn^{1+1/k})$ bits of storage [26].

Somewhat better results are known for unweighted graphs, where the approximation often has an additive term in addition to the multiplicative term. More precisely, if $d_G(s,t)$ denotes the actual distance between *s* and *t* in *G*, then an $(\alpha,\beta )$*-approximate oracle* reports a distance $d^*$ such that $d_G(s,t) \leqslant d^* \leqslant \alpha \cdot d_G(s,t)+\beta$. Patrascu and Roditty [24] presented a (2, 1)-approximate oracle with *O*(1) query time that uses $O(n^{5/3})$ storage, Abraham and Gavoille [1] presented a $(2k-2,1)$-approximate oracle with *O*(*k*) query time that uses $\tilde{O}(n^{1+2/(2k-1)})$ storage (for $k\geqslant 2$).

To summarize, for non-planar graphs all distance oracles with subquadratic storage have a multiplicative error of at least 2, even in the unweighted case. The only exception is for the rather restricted case of unit-disk graphs, where Gao and Zhang [16], and later Chan and Skrepetos [6], presented a $(1+\varepsilon )$-approximate distance oracle with *O*(1) query time that uses $O_{\varepsilon }(n\log n)$ storage. (This result actually also works in the weighted setting, where the weight of an edge between two disks is the Euclidean distance between their centers.)

## A Clique-Based Separator for Geodesic Disks in $\mathbb {R}^2$

Let *d* be a metric defined on a closed path-connected subset $F\subset \mathbb {R}^2$, and let $\mathcal {D}=\{D_1,\ldots,D_n\}$ be a set of geodesic disks in *F*, with respect to the metric *d*. Thus each disk^5^ $D_i$ is defined as $D_i:=\{ q\in F: d(q,p_i) \leqslant r_i\}$, where $p_i\in F$ is the center of $D_i$ an $r_i\geqslant 0$ is its radius. Let $\mathcal {D}_0:=\mathcal {D}$ and recall that $\mathcal {G}^{\times }(\mathcal {D}_0)$ denotes the intersection graph of $\mathcal {D}_0$. (The reason for introducing the notation $\mathcal {D}_0$ will become clear shortly.) We denote the set of edges of $\mathcal {G}^{\times }(\mathcal {D}_0)$ by *E* and define $m:=|E|$.

### A First Bound

Our basic construction of a clique-based separator $\mathcal {S}$ for $\mathcal {G}^{\times }(\mathcal {D}_0)$, yields a separator with $O(n^{4/5})$ cliques. In Sect. 2.2, we will apply a bootstrapping scheme to further reduce the size of the separator. The basic construction proceeds in three steps: in a preprocessing step we reduce the ply of the set of disks we need to deal with, in the second step we prove that if the ply is sublinear then the number of edges in the intersection graph is subquadratic, and in the third step we construct the separator.

**Step 1: Reducing the ply.** For a point $p\in F$, let $\mathcal {D}_0(p):=\{D_i \in \mathcal {D}_0: p\in D_i\}$ be the set of disks from $\mathcal {D}_0$ containing *p*—note that $\mathcal {D}_0(p)$ forms a clique in $\mathcal {G}^{\times }(\mathcal {D}_0)$—and define ${{\,\textrm{ply}\,}}(p):=|\mathcal {D}_0(p)|$ to be the ply of *p* with respect to $\mathcal {D}_0$. The ply of the set $\mathcal {D}_0$ is defined as ${{\,\textrm{ply}\,}}(\mathcal {D}_0):=\max \{p\in F: {{\,\textrm{ply}\,}}(p)\}$.

We start by reducing the ply of $\mathcal {D}_0$ in the following greedy manner. Let $\alpha$ be a fixed constant with $0<\alpha <1$. In the basic construction we will use $\alpha =1/5$, but in our bootstrapping scheme we will work with other values as well. We check whether there exists a point *p* such that $|\mathcal {D}_0(p)|\geqslant \frac{1}{4}n^{\alpha }$. If so, we remove $\mathcal {D}_0(p)$ from $\mathcal {D}_0$ and add it as a clique to $\mathcal {S}$. We repeat this process until ${{\,\textrm{ply}\,}}(\mathcal {D}_0)<\frac{1}{4}n^{\alpha }$. Thus in the first step at most $4n^{1-\alpha }$ cliques are added to $\mathcal {S}$.

To avoid confusion with our initial set $\mathcal {D}_0$, we denote the set of disks remaining at the end of Step 1 by $\mathcal {D}_1$ and we denote the set of edges of $\mathcal {G}^{\times }(\mathcal {D}_1)$ by $E_1$.

**Step 2: Bounding the size of**$E_1$. To bound the size of $E_1$, we draw a shortest path $\pi _{ij}$ between the centers $p_i,p_j$ of every two intersecting disks $D_i,D_j\in \mathcal {D}_1$, thus obtaining a geometric realization of the graph $\mathcal {G}^{\times }(\mathcal {D}_1)$. From now on, with a slight abuse of notation, we will not distinguish between an edge $(D_i,D_j)$ in $\mathcal {G}^{\times }(\mathcal {D}_1)$ and its geometric realization $\pi _{ij}$. Let $\Pi (\mathcal {D}_1):=\{ \pi _{ij}: (D_i,D_j) \in E_1\}$ be the resulting set of paths. To focus on the main idea behind our proof we will, for the time being, assume that $\Pi (\mathcal {D}_1)$ is a *proper path set*: a set of paths such that any two paths $\pi _{ij},\pi _{k\ell }$ have at most two points in common, each intersection point is either a shared endpoint or a proper crossing, and no proper crossing coincides with another proper crossing or with an endpoint. When $\pi _{ij}$ and $\pi _{k\ell }$ have a proper crossing, we say that they *cross* and otherwise, they do not cross. In Sect. 2.3, we will argue that the proof still goes through without assuming that $\Pi (\mathcal {D}_1)$ is a proper path set, assuming that our shortest-path metric *d* is *well-behaved* (as defined in Sect. 2.3).

We need the following well-known result about the number of crossings in dense graphs, known as the Crossing Lemma [2, 21]. The term *planar drawing* in the Crossing Lemma refers to a drawing where no edge interior passes through a vertex and all intersections are proper crossings. Thus it applies to a proper path set.

#### Lemma 2.1

(Crossing Lemma) There exists a constant $c>0$, such that every planar drawing of a graph with *n* vertices and $m\geqslant 4n$ edges contains at least $c\frac{m^3}{n^2}$ crossings.

Using the Crossing Lemma we will show that $|E_1|=O(n^{\frac{3+\alpha }{2}})$, as follows. If $|E_1|=O(n^{\frac{3+\alpha }{2}})$ does not hold, then by the Crossing Lemma there must be many crossings between the edges $\pi _{ij}\in E_1$. We will show that this implies that there is a crossing of ply greater than $\frac{1}{4}n^{\alpha }$, thus contradicting that ${{\,\textrm{ply}\,}}(\mathcal {D}_1)<\frac{1}{4}n^{\alpha }$. We now make this idea precise.

#### Lemma 2.2

Let $\mathcal {G}^{\times }(\mathcal {D}_1)=(\mathcal {D}_1,E_1)$ be the intersection graph of a set $\mathcal {D}_1$ of disks such that ${{\,\textrm{ply}\,}}(\mathcal {D}_1)<\frac{1}{4}n^{\alpha }$. Then $|E_1|\leqslant \sqrt{\frac{4}{c}} \cdot n^{\frac{3+\alpha }{2}}$, where *c* is the constant appearing in the Crossing Lemma.

**Fig. 1:**
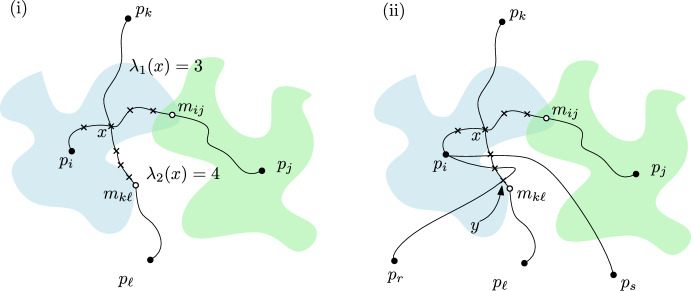
**i** An example of a labeling for a crossing $x\in \mathcal {X}$. **ii** Here, the crossing $y\in \pi _{ij}[x,m_{k\ell }]$ is assigned to $D_i$ a total of four times

#### Proof

Consider the proper path set $\Pi (\mathcal {D}_1)$ and let $\mathcal {X}$ be the set of crossings between the paths in $\Pi (\mathcal {D}_1)$. Assume for a contradiction that $|E_1|>\sqrt{\frac{4}{c}}\cdot n^{\frac{3+\alpha }{2}}$. We will show that then there has to exist a crossing $x\in \mathcal {X}$ of ply at least $\frac{n^{\alpha }}{4}$, which contradicts that ${{\,\textrm{ply}\,}}(\mathcal {D}_1)<\frac{1}{4}n^{\alpha }$.

We start by giving a lower bound on the total ply of all crossings in the drawing. To this end, we split each edge $\pi _{ij}\in E_1$ in two *half-edges* as follows. For two points $x,y\in \pi _{ij}$, let $\pi _{ij}[x,y]$ denote the subpath of $\pi _{ij}$ between *x* and *y*. Recall that $p_i$ is the center of disk $D_i$. We now pick an arbitrary point $m_{ij} \in \pi _{ij} \cap \left( D_i\cap D_j \right)$ and split $\pi _{ij}$ at $m_{ij}$ into a half-edge $\pi _{ij}[p_i,m_{ij}]$ connecting $p_i$ to $m_{ij}$ and a half-edge $\pi _{ij}[p_j,m_{ij}]$ connecting $p_j$ to $m_{ij}$. For brevity, we will denote these two half-edges by $h_{ij}$ and $h_{ji}$, respectively. Clearly, each half-edge has length at most the radius of the disk it lies in, and so $h_{ij}\subset D_i$ and $h_{ji}\subset D_j$. We denote the resulting set of half-edges by $\mathcal {E}_1$.

We label each crossing $x\in \mathcal {X}$ with an unordered pair of integers $\{\lambda _1(x),\lambda _2(x)\}$, defined as follows: if *x* is the crossing between the half-edges $h_{ij},h_{k\ell }$, then $\lambda _1(x)$ is the number of crossings contained in $\pi _{ij}[x,m_{ij}]$ and $\lambda _2(x)$ is the number of crossings contained in $\pi _{k\ell }[x,m_{k\ell }]$; see Fig. 1(i). This labeling is useful to obtain a rough bound on the total ply of all crossings, because of the following observation, which immediately follows from the triangle inequality.

#### Observation 1

Consider a crossing $x= h_{ij} \cap h_{k\ell }$. If $d(x,m_{k\ell })\leqslant d(x,m_{ij})$ then all crossings $y\in \pi _{k\ell }[x,m_{k\ell }]$ are contained in $D_i$, and otherwise all crossings $y\in \pi _{ij}[x,m_{ij}]$ are contained in $D_k$.

Let $K:=\sum _{x\in \mathcal {X}} {{\,\textrm{ply}\,}}(x)$ denote the total ply of all crossings. The following claim bounds *K* in terms of the labels $\{\lambda _1(x),\lambda _2(x)\}$.

#### Claim 1

$$K > \frac{1}{2|\mathcal {D}_1|} \sum _{x\in \mathcal {X}} \min \{\lambda _1(x),\lambda _2(x)\}$$.

#### Proof

Define $K(D_i):=\left| \{ x\in \mathcal {X}: x\in D_i \}\right|$ to be the contribution of $D_i$ to the total ply *K*, and note that

$$ K = \sum _{x\in \mathcal {X}} {{\,\textrm{ply}\,}}(x) = \sum _{x\in \mathcal {X}} \left| \{ D_i \in \mathcal {D}_1: x\in D_i \}\right| = \sum _{D_i \in \mathcal {D}_1} \left| \{ x\in \mathcal {X}: x\in D_i \}\right| = \sum _{D_i\in \mathcal {D}_1} K(D_i). $$

Now consider a crossing $x=h_{ij}\cap h_{k\ell }$. If $d(x,m_{k\ell })\leqslant d(x,m_{ij})$ then we assign *x* to $D_i$, and otherwise we assign *x* to $D_k$. Let $\mathcal {X}(D_i)$ be the set of crossings assigned to $D_i$. By Observation 1 and the definition of the label $\{\lambda _1(x),\lambda _2(x)\}$, the disk $D_i$ contains at least $\min \{\lambda _1(x),\lambda _2(x)\}$ crossings $y\in \pi _{k\ell }[x,m_{k\ell }]$, for any $x=h_{ij}\cap h_{k\ell }\in \mathcal {X}(D_i)$. Thus, summing over all crossings $x\in \mathcal {X}(D_i) \cap h_{ij}$ and all half-edges $h_{ij}$ incident to $D_i$, we find that

$$ K(D_i) \geqslant \frac{1}{2\deg (D_i)} \sum _{h_{ij}} \sum _{x\in \mathcal {X}(D_i)\cap h_{ij}} \min \{\lambda _1(x),\lambda _2(x)\} > \frac{1}{2|\mathcal {D}_1|} \sum _{x\in \mathcal {X}(D_i)} \min \{\lambda _1(x),\lambda _2(x)\}, $$

where $\deg (D_i)$ denotes the degree of $D_i$ in $\mathcal {G}^{\times }(\mathcal {D}_1)$. The factor $\frac{1}{2\deg (D_i)}$ arises because a crossing $y\in h_{k\ell }$ can be counted up to $2\deg (D_i)$ times in the expression $\sum _{x\in \mathcal {X}(D_i)} \min \{\lambda _1(x),\lambda _2(x)\}$, namely at most twice for every half-edge incident to $D_i$; see Fig. 1(ii). (Twice, because a pair of paths in a proper path set may cross twice.) Since each crossing is assigned to exactly one set $\mathcal {X}(D_i)$, we obtain

$$\begin{aligned} K&= \sum _{D_i\in \mathcal {D}_1} K(D_i) > \sum _{D_i\in \mathcal {D}} \left( \frac{1}{2|\mathcal {D}_1|} \sum _{x\in \mathcal {X}(D_i)} \min \{\lambda _1(x),\lambda _2(x)\} \right) \\&= \frac{1}{2|\mathcal {D}_1|} \sum _{x\in \mathcal {X}} \min \{\lambda _1(x),\lambda _2(x)\}. \end{aligned}$$

$$\square $$

From the Crossing Lemma and our initial assumption that $|E_1|>\sqrt{\frac{4}{c}}\cdot n^{\frac{3+\alpha }{2}}$, we have that

1 $$\begin{aligned}|\mathcal {X}| \geqslant c \frac{|E_1|^3}{n^2} >4|E_1| n^{1+\alpha } = 2|\mathcal {E}_1| n^{1+\alpha }. \end{aligned}$$

In order to get a rough bound for $\sum _{x\in \mathcal {X}} \min \{\lambda _1(x),\lambda _2(x)\}$, we will ignore crossings with small labels, while ensuring that we don’t ignore too many crossings in total. More precisely, for every half-edge $h_{ij}$, we disregard its first $n^{1+\alpha }$ crossings, starting from the one closest to $m_{ij}$. We let $\mathcal {X}^*$ denote the set of remaining crossings. Note that $|\mathcal {X}^*| \geqslant|\mathcal {X}|-|\mathcal {E}_1|n^{1+\alpha }$, and $\min \{\lambda _1(x),\lambda _2(x)\} \geqslant n^{1+\alpha }$ for every $x\in \mathcal {X}^*$. Therefore

$$ K > \frac{1}{2|\mathcal {D}_1|} \sum _{x\in \mathcal {X}^* } \min \{\lambda _1(x),\lambda _2(x)\} = \frac{1}{2|\mathcal {D}_1|} \cdot|\mathcal {X}^*| \cdot n^{1+\alpha } \geqslant \frac{\left(|\mathcal {X}|-|\mathcal {E}_1|n^{1+\alpha }\right) n^{1+\alpha }}{2|\mathcal {D}_1|} \geqslant \frac{|\mathcal {X}|}{4}n^{\alpha }. $$

This means that there exists a crossing $x\in \mathcal {X}$ of ply at least $\frac{1}{4}n^{\alpha }$, which contradicts the condition of the lemma and thus finishes the proof. $\square$

**Step 3: Applying the separator theorem for string graphs.** Lee’s separator theorem for string graphs [20] states that any string graph on *m* edges admits a balanced separator of size $O(\sqrt{m})$. It is well known (and easy to show) that any intersection graph of path-connected sets in the plane is a string graph. Hence, we can apply Lee’s result to $\mathcal {G}^{\times }(\mathcal {D}_1)$ which, as we have just shown, has $O(n^{\frac{3+\alpha }{2}})$ edges. Thus, $\mathcal {G}^{\times }(\mathcal {D}_1)$ has a separator of size $O(n^{\frac{3+\alpha }{4}})$. If we add the vertices of this separator as singletons to our clique-based separator $\mathcal {S}$, then, together with the cliques added in Step 1, we obtain a separator consisting of $O(n^{\frac{3+\alpha }{4}} + n^{1-\alpha })$ cliques. Picking $\alpha =1/5$, and anticipating the extension to the case where $\Pi (\mathcal {D}_1)$ is not a proper path set, we obtain the following result.

#### Proposition 2.3

Let *d* be a well-behaved shortest-path metric on a closed and path-connected subset $F\subset \mathbb {R}^2$ and let $\mathcal {D}$ be a set of *n* geodesic disks with respect to the metric *d*. Then $\mathcal {G}^{\times }(\mathcal {D})$ has a balanced clique-based separator consisting of $O(n^{4/5})$ cliques.

### Bootstrapping

We now describe a bootstrapping mechanism to improve the size of our clique-based separator. We first explain how to apply the mechanism once, to obtain a clique-based separator consisting of $O(n^{10/13})$ cliques. Then we apply the method repeatedly to obtain a separator consisting of $O(n^{3/4+\varepsilon })$ cliques.

**The basic bootstrapping mechanism.** The idea of our bootstrapping mechanism is as follows. After reducing the ply in Step 1, we argued that the number of remaining edges is subquadratic. We can actually reduce the number of edges and crossings even further, by reducing the maximum degree of the graph $\mathcal {G}^{\times }(\mathcal {D}_1)$. To this end, after reducing the ply, we remove from $\mathcal {G}^{\times }(\mathcal {D}_1)$ all vertices $D_i$ such that $\deg (D_i)\geqslant n^\beta$, for some constant $0<\beta <1$ whose value will be determined later. All these high degree vertices are placed in our clique-based separator as singletons. If *h* is the number of these vertices, then $h\cdot n^\beta \leqslant 2|E_1|$ and so $h= O( n^{\frac{3+\alpha }{2}-\beta })$. We denote the resulting graph by $\mathcal {G}^{\times }(\mathcal {D}_2) = (\mathcal {D}_2,E_2)$ and let $\mathcal {E}_2$ be the half-edges corresponding to the edges in $E_2$.

To minimize the number of cliques in our separator, the constants $\alpha$ and $\beta$ should be chosen to satisfy the equation

2 $$\begin{aligned} \frac{3+\alpha }{2}-\beta =1-\alpha. \end{aligned}$$

Next, we show that after reducing the maximum degree, the number of edges has also decreased.

#### Lemma 2.4

For the graph $\mathcal {G}^{\times }(\mathcal {D}_2)$ defined as above, it holds that $|E_2| \leqslant \sqrt{\frac{4}{c}} n^{1+\frac{\alpha +\beta }{2}}$, where *c* is the constant appearing in the Crossing Lemma.

#### Sketch of proof

The proof is essentially the same as the proof of Lemma 2.2, so we only mention the key differences. For completeness, we provide the full proof in the Appendix. We assume for a contradiction that $|E_2| > \sqrt{\frac{4}{c}} n^{1+\frac{\alpha +\beta }{2}}$. Then, we lower bound the number of crossings (denoted now by $\mathcal {X}_2$) as

$$|\mathcal {X}_2| \geqslant c \frac{|E_2|^3}{n^2} >2|\mathcal {E}_2|n^{\alpha +\beta }. $$

We then proceed with the same labeling procedure as in Step 2 in order to lower bound the total ply $K_2$ of all crossings. A key difference is that now we only need to divide by $2n^\beta$, since $n^\beta$ is the maximum degree of $\mathcal {G}^{\times }(\mathcal {D}_2)$:

$$ K_2 \geqslant \frac{1}{2n^\beta } \sum _{x\in \mathcal {X}_2} \min \{\lambda _1(x),\lambda _2(x)\}. $$

To get an estimate for $K_2$, we remove from every half-edge, its first $n^{\alpha +\beta }$ crossings. In this way there are at least $|\mathcal {X}_2|-|\mathcal {E}_2| n^{\alpha +\beta }$ crossings remaining, each having a minimum label larger than $n^{\alpha +\beta }$. Therefore

$$ K_2> \frac{\left(|\mathcal {X}_2|-|\mathcal {E}_2|n^{\alpha +\beta }\right) n^{\alpha +\beta }}{2n^\beta } > \frac{|\mathcal {X}_2|}{4}n^{\alpha } $$

so we again get a contradiction, since there has to exist a crossing with ply at least $\frac{1}{4}n^{\alpha }$. $\square$

Step 3 now gives us that $\mathcal {G}^{\times }(\mathcal {D}_2)$ has a separator of size $O(n^{\frac{2+\alpha +\beta }{4}})$, whose vertices we add to our clique-based separator as singletons. In order to minimize the number of cliques in our separator, we now have the equation

3 $$\begin{aligned} \frac{2+\alpha +\beta }{4}=1-\alpha. \end{aligned}$$

Solving the system of Equations (2) and (3) gives $\alpha =3/13$ and $\beta =11/13$. This solution corresponds to a clique-based separator of size $O(n^{10/13})$.

**Repeated bootstrapping.** The method we just described can be applied repeatedly: After reducing the maximum degree to $n^{\beta }$, we showed that $|E_2|=O(n^{1+\frac{\alpha +\beta }{2}})$. This allows us to reduce the maximum degree even more, by placing vertices of degree at least $n^\gamma$ in the clique-based separator, for some $\gamma <\beta$. If we repeat this process $O(\log (1/\varepsilon ))$ times, we can obtain a clique-based separator of size $O(n^{3/4+\varepsilon })$, as is shown in the following theorem.

#### Theorem 2.5

Let *d* be a well-behaved shortest-path metric on a closed and path-connected subset $F\subset \mathbb {R}^2$, and let $\mathcal {D}$ be a set of *n* geodesic disks with respect to the metric *d*. Then $\mathcal {G}^{\times }(\mathcal {D})$ has a balanced clique-based separator consisting of $O(n^{\frac{3}{4}+\varepsilon })$ cliques.

#### Proof

Let $\mathcal {S}$ denote our clique-based separator. As before, we start by reducing the ply of the subdivision, by finding points of ply at least $\frac{n^{\alpha _1}}{4}$, for some constant $0<\alpha _1<1$, and placing the corresponding cliques into $\mathcal {S}$. Our separator now has a size of $O(n^{1-\alpha _1})$. Let $\mathcal {D}_1$ denote the remaining set of disks and let $\mathcal {G}^{\times }(\mathcal {D}_1)=(\mathcal {D}_1,E_1)$ denote the resulting subgraph after this step. Next, we repeatedly apply the following procedure for $i=2,\dotsc,k$: we reduce the maximum degree of $\mathcal {G}^{\times }(\mathcal {D}_{i-1})$ to $n^{\alpha _i}$ (for some positive constant $\alpha _i< \alpha _{i-1}$) by removing from $\mathcal {G}^{\times }(\mathcal {D}_{i-1})$ vertices of degree at least $n^{\alpha _i}$ and we place them in $\mathcal {S}$ as singletons. We denote by $\mathcal {D}_i$ the remaining set of disks and let $\mathcal {G}^{\times }(\mathcal {D}_{i})=(\mathcal {D}_i,E_i)$ be the resulting subgraph.

Lemma 2.4 gives us that $|E_2| \leqslant \sqrt{\frac{4}{c}} n^{1+\frac{\alpha _{1}+\alpha _2}{2}}$, for $i=2,\dotsc,k$. In exactly the same way we can show that $|E_{i}| \leqslant \sqrt{\frac{4}{c}} n^{1+\frac{\alpha _1+\alpha _{i}}{2}}$, for $i=3,...,n$.

During the above procedure, we have introduced *k* unknown constants $\alpha _1,\alpha _2,\dotsc,\alpha _k$ and so we need *k* equations in order to calculate their values. To this end, let $h_{i}$ denote the number of vertices of $G_i$ with degree at least $n^{\alpha _{i+1}}$, for $i=1,\dotsc,{k-1}$. Then, due to Lemma 2.2, we have $h_{1} \cdot n^{\alpha _{2}}\leqslant 2|E_1|,$ and so

$$ h_1 = O(n^{\frac{3+\alpha _1}{2}-\alpha _2}). $$

For $i=2,...,k-1$ we have $h_{i} \cdot n^{\alpha _{i+1}}\leqslant 2|E_i|,$ and so

$$ h_i = O(n^{1+\frac{\alpha _1+\alpha _i}{2}-\alpha _{i+1}}). $$

In the last step, we are left with the graph $\mathcal {G}^{\times }(\mathcal {D}_k)$, for which we have that $|E_k| \leqslant \sqrt{\frac{4}{c}} n^{1+\frac{\alpha _{1}+\alpha _k}{2}}$. We now apply the separator result on string graphs, which states that any string graph with *m* edges has a separator of size $O(\sqrt{m})$. Thus $\mathcal {G}^{\times }(\mathcal {D}_k)$ has a separator of size $O(n^{\frac{2+\alpha _{1}+\alpha _k}{4}})$ whose vertices we also place in $\mathcal {S}$. As a result, the size of our clique-based separator is given by

$$|\mathcal {S}| = O \left( n^{1-\alpha _1}+ \sum _{i=1}^{k-1} h_k + n^{\frac{2+\alpha _{1}+\alpha _k}{4}} \right) $$

which gives the following system of *k* equations:

$$\begin{aligned} 1-\alpha _1&= \frac{3+\alpha _1}{2}-\alpha _2 \qquad \qquad (4.1) \\ 1-\alpha _1&= 1+\frac{\alpha _1+\alpha _i}{2}-\alpha _{{i+1}} \quad \text {for} \quad i=2,3,...,k-1 \qquad \qquad (4.i)\\ 1-\alpha _1&= \frac{2+\alpha _{1}+\alpha _k}{4} \qquad \qquad (4.k) \end{aligned}$$

This system can be rewritten as

$$\begin{aligned} 3\alpha _1&= 2\alpha _2 -1 \qquad \qquad (4.1)\\ 3\alpha _1&= 2\alpha _{i+1}-\alpha _i \quad \text {for} \quad i=2,3,...,k-1 \qquad \qquad (4.i) \\ 5\alpha _1&= 2-\alpha _k \qquad \qquad (4.k) \end{aligned}$$

By multiplying the *i*-th equation above with $2^{i-1}$ and summing up, we can see that the terms $\alpha _2,...,\alpha _k$ cancel and we are left with the equation

$$ \alpha _1\left( 3\sum _{i=0}^{k-2} 2^i+5\cdot 2^{k-1}\right) = 2^k-1, $$

which solves to

$$ \alpha _1 = \frac{2^k-1}{2^{k+2}-3}. $$

Thus $1-\alpha _1 = \frac{3\cdot 2^k -2}{4\cdot 2^k -3}$ (which converges to $\frac{3}{4}$ as $k\rightarrow \infty$). By choosing^6^$k= \log _2(1/\varepsilon )$, we have

$$ 1-\alpha _1 = \frac{3-2\varepsilon }{4-3\varepsilon }, $$

which is smaller than $\frac{3}{4}+\varepsilon$ for $\varepsilon <1$. Therefore, for these choices of $\alpha _1$ and *k*, our clique-based separator has a size of $O(n^{\frac{3}{4}+\varepsilon })$ as claimed. $\square$

De Berg et al. [12] showed that if a class of graphs admits a clique-based separator consisting of *S*(*n*) cliques, and the separator can be constructed in polynomial time, then one can solve *q*-Coloring in $2^{O(S(n))}$ time. Note that if we can efficiently compute the boundaries $\partial D_i$,^7^ then we can also compute our separator in polynomial time. Indeed, we can then efficiently compute $\mathcal {G}^{\times }(\mathcal {D})$ and the arrangement of the disk boundaries, which allows us to perform Step 1 (reducing the ply) in polynomial time. Since there is a polynomial-time algorithm for computing a separator for string graphs [20], this is easily seen to imply that the separator construction runs in polynomial time. Thus we obtain the following result.

#### Corollary 2.6

Let *d* be a shortest-path metric on a connected subset $F\subset \mathbb {R}^2$ and let $\mathcal {D}$ be a set of *n* geodesic disks with respect to the metric *d*, where *d* is such that the boundaries $\partial D_i$ of the disks in $\mathcal {D}$ can be computed in polynomial time. Let $q\geqslant 1$ and $\varepsilon >0$ be fixed constants. Then *q*-Coloring can be solved in $2^{O(n^{\frac{3}{4}+\varepsilon })}$ time on $\mathcal {G}^{\times }(\mathcal {D})$.

### Obtaining a Proper Path System

So far we assumed that $\Pi (\mathcal {D})$ is a proper path set. In general this may not be the case, since the paths in $\Pi (\mathcal {D})$ may overlap along 1-dimensional subpaths and a pair of paths can meet multiple times. (The latter can happen since shortest paths need not be unique). Lemma 2.7 will allow us to still work with a proper path set in our proof. The proof of this lemma requires two technical assumptions on our shortest-path metric *d*. We thus now introduce the concept of a *well-behaved shortest-path metric*.

**Well-behaved shortest-path metrics.** We assume that for any finite set $P=\{ p_1,\ldots,p_n\}$ of points in *F*, there exists a set $\Pi (P) = \{ \pi _{ij}: 1 \leqslant i<j \leqslant n \}$ of shortest paths between the points in *P* with the following properties:

1. For any two paths $\pi _{ij},\pi _{k\ell }\in \Pi (P)$, the intersection $\pi _{ij}\cap \pi _{k\ell }$ consists of finitely many connected components.
2. Let $P^+\supset P$ be the set of endpoints of these components, and let $\Gamma _{ij}$ be the set of subpaths into which $\pi _{ij}$ is partitioned by the points in $P^+$. Note that any two subpaths $\gamma \in \Gamma _{ij}$ and $\gamma'\in \Gamma _{k\ell }$ either have disjoint interiors or they are identical. Let $\Gamma = \bigcup _{i,j} \Gamma _{ij}$ be the set of all distinct subpaths. Then there are values $\delta _1,\delta _2>0$ such that the following holds.
  For any $v\in P^+$, the Euclidean ball $B(v,\delta _1)$ of radius $\delta _1$ centered at *v* only intersects paths from $\Pi (P)$ that contain *v*, and inside $B(v,\delta _1)$ any two paths are either disjoint (except at *v*) or they coincide. Moreover, the balls $B(v,\delta _1)$ for $v\in P^+$ are pairwise disjoint.
  Outside the balls $B(v,\delta _1)$, the minimum distance between any two subpaths $\gamma,\gamma'\in \Gamma$ is at least $\delta _2$.

We call a metric satisfying these conditions *well-behaved*. The conditions are easily seen to be satisfied when *F* is a closed and connected polygonal region and the paths $\pi _{ij}$ are shortest (in the Euclidean sense) paths inside *F*. In fact, *F* does not have to be polygonal; its boundary may consist of finitely many constant-degree algebraic curves. Another example is when the paths in $\pi _{ij}$ are projections onto $\mathbb {R}^2$ of shortest paths on a polyhedral terrain, or when the paths are shortest paths in a weighted polygonal subdivision. (In the latter case, the length of a section of the path inside a region is multiplied by the weight of the region [23]. In this setting, shortest paths are piece-wise linear and so they satisfy the conditions.)

We can now proceed with the proof of the lemma. It might be known, but we have not been able to find a reference. Recall that *E* is the edge set of $\mathcal {G}^{\times }(\mathcal {D})$.

#### Lemma 2.7

Let *d* be a well-behaved shortest-path metric on a closed and path-connected subset $F\subset \mathbb {R}^2$, and let $\Pi (\mathcal {D}) = \{ \pi _{ij}: (D_i,D_j)\in E\}$ be a set of shortest paths with the properties stated above. Then there is a proper path set $\Pi'(\mathcal {D}) = \{\pi'_{ij}: (D_i,D_j)\in E\}$ in $\mathbb {R}^2$ with the following property: for every pair $\pi'_{ij},\pi'_{k\ell }$ of crossing paths in $\Pi'(\mathcal {D})$, the corresponding paths $\pi _{ij},\pi _{k\ell }$ in $\Pi (\mathcal {D})$ intersect.

#### Proof

We will prove the lemma in two steps. First, we modify the paths such that any two paths $\pi _{ij}$ and $\pi _{k\ell }$ meet in a single connected component, and then we perturb these paths slightly to obtain a set $\Pi'(\mathcal {D})$ where any two paths cross in at most two points.

**Fig. 2:**
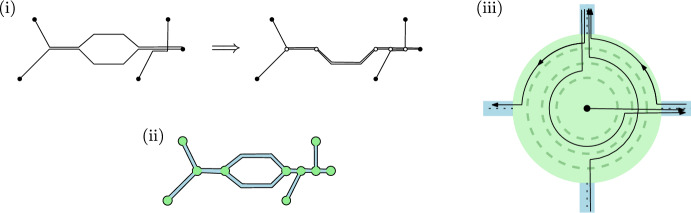
**i** The input paths (left) and the graph $\mathcal {G}^+$ after re-routing the paths (right). **ii** The thickened graph. **iii** Drawing the paths inside a thick vertex

The first modification is done as follows. For each pair $\pi _{ij},\pi _{k\ell }\in \Pi (\mathcal {D})$, and for each connected component $I\subset \pi _{ij}\cap \pi _{k\ell }$, we add both endpoints of *I* to the set *P*, thus (by the first property of well-behaved shortest-path metrics) obtaining a finite set $P^+$. The set $P^+$, together with the pieces of the paths $\pi _{ij}$ connecting them, forms a plane graph $\mathcal {G}^+=(P^+,E^+)$. We now slightly modify the edge lengths in $\mathcal {G}^+$, to ensure that there is a unique shortest path between any two points in $P^+$, and we replace each original path $\pi _{ij}$ by the shortest path between $p_i$ and $p_j$ in $\mathcal {G}^+$; see Fig. 2(i) for an example.

Since the shortest paths between any two vertices in $\mathcal {G}^+$ are unique, two paths $\pi _{ij},\pi _{k\ell }$ do not intersect in more than one connected component. Moreover, the new paths are still shortest paths under the original metric. Formally, the perturbation of the edges lengths is done as follows. Let $e_1,e_2,...,e_k$ be any ordering on the edges of $\mathcal {G}^+$. Then, perturb the edge lengths such that the new lengths are given by $|e_i'|=|e_i|+2^i\varepsilon$, for an arbitrarily small $\varepsilon >0$. Any path that was shortest initially, will remain shortest and no two paths have the same length.

Next we show how to slightly perturb the paths such that all crossings are proper. For that, we will make use of the second property of well-behaved shortest-path metrics to thicken the graph $\mathcal {G}^+$. Intuitively, we replace the edges of $\mathcal {G}^+$ by thick curves and its vertices by small balls. Within each thick curve, we can slightly separate all paths that use the corresponding edge. Curves going to the same vertex meet at the disk around it. We then show that it’s possible to route paths within these balls, such that paths only possibly cross within the balls where they meet for the first time or for the last time. Formally this is done as follows.

Let $\delta _1,\delta _2>0$ be the values specified in the second property of well-behaved metrics. We thicken $\mathcal {G}^+$ by replacing each vertex $v\in P^+$ by the ball $B(v,\delta _1)$—we call this ball a *thick vertex*—and by replacing each edge in $\mathcal {G}^+$ by a *thick edge* of width $2\delta _2/3$. (Formally, we take the Minkowski sum of the original edge and a ball of radius $\delta /3$, and remove the parts of the resulting thick edge inside the balls $B(v,\delta _1)$.) See Fig. 2(ii) for an example. Note that the thick edges are pairwise disjoint.

Now we draw the paths $\pi _{ij}\in \Pi'(\mathcal {D})$ one by one, as follows. We start at the point $p_i$ and follow the thick edges of $\pi _{ij}$ until we reach $p_{ij}$. An invariant of our process is that the paths may only cross inside thick vertices, and not inside thick edges. It is easy to see that this invariant can be maintained: when $\pi _{ij}$ enters a thick edge in between two previously drawn paths, then we can stay in between these two paths until we reach the next thick vertex. It will be convenient to view the edges as roads with two lanes, where we will always stay in the right lane as we draw the paths. (Thus the lanes that we use depend on whether we draw $\pi _{ij}$ from $p_i$ to $p_j$ or in the other direction; this choice can be made arbitrarily.)

Routing inside a thick vertex $B(v,\delta _1)$ is done as follows. Let $\deg (v)$ denote the degree of vertex *v* in $\mathcal {G}^+$. We view $B(v,\delta _1)$ as a roundabout with $\deg (v)-1$ lanes $L_1,\ldots,L_{\deg (v)-1}$, where $L_1$ is the outermost lane. Now suppose we draw a path $\pi _{ij}$ and we enter the roundabout through some thick edge. Then draw $\pi _{ij}$ as follows: if $\pi _{ij}$ leaves the roundabout at the *j*-th exit in counterclockwise order (counting from the road on which we enter) then we will use lane $L_j$ on the roundabout to draw $\pi _{ij}$; see Figure 2(iii) for some examples. We can easily do this in such a way that we maintain the following invariant: paths entering and exiting the roundabout on the same roads do not cross. Note that paths that use the same two edges incident to *v* but that were drawn in the opposite direction, do not cross either. Thus two paths $\pi _{ij},\pi _{k\ell }$ may only cross when *v* is either the first or the last vertex of $\pi _{ij}\cap \pi _{k\ell }$, and when this happens they have a single crossing inside $B(v,\delta _1)$. Hence, two paths have at most two crossings and these crossings are proper crossing.

There is one thing that we have swept under the rug so far, namely what happens at the start vertex (or end vertex) of the path $\pi _{ij}$. In this case we simply draw $\pi _{ij}$ from $p_i$ to the correct exit road (or we draw $\pi _{ij}$ from the entry road to $p_j$). This way we may have to create intersection with paths $\pi _{kl}$ traversing the roundabout, but this is okay since this is then the first (or last) vertex where $\pi _{ij}$ meets $\pi _{kl}$. $\square$

Recall that the assumption that $\Pi (\mathcal {D})$ is a proper path set was used so that we could apply the Crossing Lemma. Now, instead of applying the Crossing Lemma to (a subset of) $\Pi (\mathcal {D})$ we apply it to (the corresponding subset of) $\Pi'(\mathcal {D})$. Then we obtain a bound on $|\mathcal {X}|$, the number of crossings of the perturbed paths, which by Lemma 2.7 gives us a collection of points on the intersections of the original paths. These points can be used as the set $\mathcal {X}$ in the proof of Lemma 2.2. Note that Observation 1 in that proof still holds. Also note that the fact that crossings may now coincide is not a problem: we just need to re-define $\lambda _1(x)$ (and, similarly, $\lambda _2(x)$) so that crossings that coincide with *x* are also counted. We conclude that the proof also works without the assumption that $\Pi (\mathcal {D})$ is a proper path set.

## Application to Distance Oracles

With our clique-based separator at hand, we can apply standard techniques to obtain an almost-exact hop-distance oracle for $\mathcal {G}^{\times }(\mathcal {D})$. This is done using a *separator tree* *T* as follows.

- Let $$\mathcal {S}$$ be a clique-based separator for $$\mathcal {G}^{\times }(\mathcal {D})$$. The root of the separator tree *T* stores, for each disk $$D_i\in \mathcal {D}$$ and each clique $$C_k\in \mathcal {S}$$, the value $$ hdist (D_i,C_k) = \min _{D_j\in C_k} hdist (D_i,D_j)$$, where $$ hdist (D_i,D_j)$$ denotes the hop-distance between $$D_i$$ and $$D_j$$ in $$\mathcal {G}^{\times }(\mathcal {D})$$. Thus we need $$O(n \cdot n^{3/4+\varepsilon })=O(n^{7/4+\varepsilon })$$ storage at the root.
- Let $\mathcal {D}_A$ and $\mathcal {D}_B$ be the two subsets into which $\mathcal {S}$ splits $\mathcal {D}\setminus \mathcal {S}$, such that $|\mathcal {D}_A|,|\mathcal {D}_B|\leqslant 2n/3$ and there are no edges between $\mathcal {D}_A$ and $\mathcal {D}_B$. We recursively construct separator trees $T_A$ for $\mathcal {D}_A$ and $T_B$ for $\mathcal {D}_B$, which become the two subtrees of the root of *T*.

The amount of storage of the structure satisfies the recurrence $S(n) = O(n^{7/4+\varepsilon })$ $+ S(n_A) + S(n_B)$, where $n_A, n_B \leqslant 2n/3$ and $n_A+n_B \leqslant n$. This solves to $S(n) = O(n^{7/4+\varepsilon })$.

To answer a query for the hop-distance between two disks $D_i,D_j$ we proceed as follows. First, we determine $Z_1:=\min _{C_k\in \mathcal {S}} \left( hdist (D_i,C_k) + hdist (C_k,D_j) \right)$. When $D_i$ and $D_j$ do not lie in the same part of the partition, we are done and report $Z_1$. Otherwise, assuming without loss of generality that $D_i,D_j\in \mathcal {D}_A$, we recursively query in $T_A$, thus obtaining a value $Z_2$, and we report $\min (Z_1,Z_2)$. Thus the query time satisfies the recurrence $Q(n) = O(n^{3/4+\varepsilon }) + Q(n_A)$, where $n_A\leqslant 2n/3$, which solves to $Q(n) = O(n^{3/4+\varepsilon })$. It is easily seen that our query reports a value $d^*$ such that $d^* \leqslant hdist (D_i,D_j) \leqslant d^* +1$.

### Theorem 3.1

Let *d* be a well-behaved shortest-path metric on a closed and path-connected subset $F\subset \mathbb {R}^2$, and let $\mathcal {D}$ be a set of *n* geodesic disks with respect to the metric *d*. Let $\varepsilon >0$ be any fixed constant. Then there is a distance oracle for $\mathcal {G}^{\times }(\mathcal {D})$ that uses $O(n^{\frac{7}{4}+\varepsilon })$ storage and that can report, for any two query disks $D_i,D_j\in \mathcal {D}$, in $O(n^{3/4+\varepsilon })$ time a value $d^*$ such that $d^* \leqslant hdist (D_i,D_j) \leqslant d^* +1$.

## Concluding Remarks

In this paper, we showed that the intersection graph of a set of geodesic disks, in any well-behaved shortest-path metric in the plane, admits a clique-based separators of sublinear size, using a method that is quite different from previously used approaches. Separators have been used extensively to obtain efficient graph algorithms, and clique-based separators have already found many uses as well. We gave two straightforward applications of our new clique-based separators, namely for *q*-Coloring and almost exact distance oracles, but we expect our separator to have more applications. More generally, we hope that our paper inspires more research on intersection graphs of geodesic disks in the general setting that we studied—or, more modestly, of geodesic disks in polygons with holes or on terrains.

An obvious open problem is to improve upon our bounds: do intersection graphs of geodesic disks admit a separator of size $o(n^{3/4})$, either in general or perhaps in the setting of polygons with holes? Our distance oracle is the first almost exact distance oracle with sublinear query time and subquadratic storage in such a general setting, but (besides our novel separator) it uses only standard techniques. It would be interesting to see if more advanced techniques can be applied to get better bounds.

## Data Availability

No datasets were generated or analysed during the current study.

## Appendix

Here we provide the complete proof of Lemma 2.4. Recall that after reducing the ply, we remove from $\mathcal {G}^{\times }(\mathcal {D}_1)$ all vertices $D_i$ such that $\deg (D_i)\geqslant n^\beta$, for some constant $0<\beta <1$. All these high-degree vertices are placed in our clique-based separator as singletons. If *h* is the number of these vertices, then $h\cdot n^\beta \leqslant 2|E_1|$ and so $h= O( n^{\frac{3+\alpha }{2}-\beta })$. We denote the resulting graph by $\mathcal {G}^{\times }(\mathcal {D}_2) = (\mathcal {D}_2,E_2)$ and let $\mathcal {E}_2$ be the half-edges corresponding to the edges in $E_2$.

### Lemma A.1

For the graph $\mathcal {G}^{\times }(\mathcal {D}_2)$ defined as above, it holds that $|E_2| \leqslant \sqrt{\frac{4}{c}} n^{1+\frac{\alpha +\beta }{2}}$, where *c* is the constant appearing in the Crossing Lemma.

### Proof

We assume for a contradiction that $|E_2| > \sqrt{\frac{4}{c}} n^{1+\frac{\alpha +\beta }{2}}$. Then, we lower bound the number of crossings (denoted now by $\mathcal {X}_2$) as

$$|\mathcal {X}_2| \geqslant c \frac{|E_2|^3}{n^2} >2|\mathcal {E}_2|n^{\alpha +\beta }. $$

We label each crossing $x\in \mathcal {X}_2$ with an unordered pair of integers $\{\lambda _1(x),\lambda _2(x)\}$, as follows: if *x* is the crossing between the half-edges $h_{ij},h_{k\ell }$, then $\lambda _1(x)$ is the number of crossings contained in $\pi _{ij}[x,m_{ij}]$ and $\lambda _2(x)$ is the number of crossings contained in $\pi _{k\ell }[x,m_{k\ell }]$ (Recall that $m_{ij}\in D_i\cap D_j$ was the point at which $\pi _{ij}$ was split into two half-edges.).

Let $K_2:=\sum _{x\in \mathcal {X}_2} {{\,\textrm{ply}\,}}(x)$ denote the total ply of all crossings. The following claim bounds $K_2$ in terms of the labels $\{\lambda _1(x),\lambda _2(x)\}$.

### Claim 1

$$K_2 > \frac{1}{2 n^{\beta }} \sum _{x\in \mathcal {X}_2} \min \{\lambda _1(x),\lambda _2(x)\}$$.

Define $K_2(D_i):=\left| \{ x\in \mathcal {X}_2: x\in D_i \}\right|$ to be the contribution of $D_i$ to the total ply $K_2$, and note that

$$ K_2 = \sum _{x\in \mathcal {X}_2} {{\,\textrm{ply}\,}}(x) = \sum _{x\in \mathcal {X}_2} \left| \{ D_i \in \mathcal {D}_2: x\in D_i \}\right| = \sum _{D_i \in \mathcal {D}_2} \left| \{ x\in \mathcal {X}_2: x\in D_i \}\right| = \sum _{D_i\in \mathcal {D}_2} K_2(D_i). $$

Now consider a crossing $x=h_{ij}\cap h_{k\ell }$. If $d(x,m_{k\ell })\leqslant d(x,m_{ij})$ then we assign *x* to $D_i$, and otherwise we assign *x* to $D_k$. Let $\mathcal {X}_2(D_i)$ be the set of crossings assigned to $D_i$. By Observation 1 of Lemma 2.2 and the definition of the label $\{\lambda _1(x),\lambda _2(x)\}$, the disk $D_i$ contains at least $\min \{\lambda _1(x),\lambda _2(x)\}$ crossings $y\in \pi _{k\ell }[x,m_{k\ell }]$, for any $x=h_{ij}\cap h_{k\ell }\in \mathcal {X}_2(D_i)$. Thus, summing over all crossings $x\in \mathcal {X}_2(D_i) \cap h_{ij}$ and all half-edges $h_{ij}$ incident to $D_i$, we find that

$$\begin{aligned} K_2(D_i)&\geqslant \frac{1}{2\deg (D_i)} \sum _{h_{ij}} \sum _{x\in \mathcal {X}_2(D_i)\cap h_{ij}} \min \{\lambda _1(x),\lambda _2(x)\}\\&= \frac{1}{2 \deg (D_i)} \sum _{x\in \mathcal {X}_2(D_i)} \min \{\lambda _1(x),\lambda _2(x)\}, \end{aligned}$$

where $\deg (D_i)$ denotes the degree of $D_i$ in $\mathcal {G}^{\times }(\mathcal {D}_2)$. The factor $\frac{1}{2\deg (D_i)}$ arises because a crossing $y\in h_{k\ell }$ can be counted up to $2\deg (D_i)$ times in the expression $\sum _{x\in \mathcal {X}_2(D_i)} \min \{\lambda _1(x),\lambda _2(x)\}$, namely at most twice for every half-edge incident to $D_i$. (Twice, because a pair of paths in a proper path set may cross twice.) Since each crossing is assigned to exactly one set $\mathcal {X}_2(D_i)$ and $\deg (D_i)< n^{\beta }$, we obtain

$$\begin{aligned} K_2&= \sum _{D_i\in \mathcal {D}_2} K_2(D_i) > \sum _{D_i\in \mathcal {D}_2} \left( \frac{1}{2 n^\beta } \sum _{x\in \mathcal {X}_2(D_i)} \min \{\lambda _1(x),\lambda _2(x)\} \right) \\&= \frac{1}{2 n^{\beta }} \sum _{x\in \mathcal {X}_2} \min \{\lambda _1(x),\lambda _2(x)\}. \end{aligned}$$

$$\square $$

In order to get a rough bound for $\sum _{x\in \mathcal {X}_2} \min \{\lambda _1(x),\lambda _2(x)\}$, we will ignore crossings with small labels, while ensuring that we don’t ignore too many crossings in total. More precisely, for every half-edge $h_{ij}$, we disregard its first $n^{\alpha +\beta }$ crossings, starting from the one closest to $m_{ij}$. We let $\mathcal {X}_2^*$ denote the set of remaining crossings. Note that $|\mathcal {X}_2^*| \geqslant|\mathcal {X}_2|-|\mathcal {E}_2|n^{\alpha +\beta }$, and $\min \{\lambda _1(x),\lambda _2(x)\} \geqslant n^{\alpha +\beta }$ for every $x\in \mathcal {X}_2^*$. Therefore

$$\begin{aligned} K_2&> \frac{1}{2 n^{\beta }} \sum _{x\in \mathcal {X}_2^* } \min \{\lambda _1(x),\lambda _2(x)\} = \frac{1}{2 n^{\beta }} \cdot|\mathcal {X}_2^*| \cdot n^{\alpha +\beta } \geqslant \frac{\left(|\mathcal {X}_2|-|\mathcal {E}_2|n^{\alpha +\beta }\right) n^{\alpha +\beta }}{2 n^{\beta }}\\&\geqslant \frac{|\mathcal {X}_2|}{4}n^{\alpha }, \end{aligned}$$

where we used that $|\mathcal {X}_2|>2|\mathcal {E}_2|n^{\alpha +\beta }$.

This means that there exists a crossing $x\in \mathcal {X}_2$ of ply at least $\frac{1}{4}n^{\alpha }$, which is a contradiction.

## Footnotes

1 Hereafter we will simply refer to such a subset as a *separator* omitting the word “balanced” for brevity. Moreover, we do not require the balance factor to be 2/3, but we allow the size of the subsets *A* and *B* to be at most $\delta n$, for some fixed constant $\delta <1$.

2 The density of a set $\mathcal {D}$ of objects in $\mathbb {R}^d$ is the maximum, over all balls $b\subset \mathbb {R}^d$, of the cardinality of the sets $\{ o\in \mathcal {D}: o\cap b\ne \emptyset$ and $\textrm{diam}(o)\geqslant \textrm{diam}(b) \}$, where $\textrm{diam}(o)$ denotes the diameter of object *o*.

3 The result generalizes to $\mathbb {R}^d$, where the bound becomes $O(\lambda ^{1/d} n^{1-1/d})$.

4 In fact, the result holds for any cost function $\gamma (x)=O(x^{\frac{1}{2} -\varepsilon })$, where $\varepsilon >0$ is a constant, but in typical applications either only the number of cliques is important, or $\gamma (x)=\log x$ suffices.

5 From now on, to simplify the terminology, we omit the adjective *geodesic* and simply use the term *disk* to refer to the objects in $\mathcal {D}$.

6 We could pick *k* even larger, say $k=\log n$, but the resulting bounds will be only marginally better and look more ugly.

7 Typically the time to do this would not only depend on *n*, but also on the complexity of *F* and the distance function *d*. For simplicity we state our results in terms of *n* only. (This, of course, puts restrictions on the complexity of *F* and *d*.)
